# Supplementary material for: Inactivation of oncogenic cAMP-specific phosphodiesterase 4D by miR-139-5p in response to p53 activation
Source: eLife. 2016 Jul 7;5:e15978. doi: 10.7554/eLife.15978 (PMC4959878; doi:10.7554/eLife.15978)
Supplement: Supplementary file 1. — DOI: http://dx.doi.org/10.7554/eLife.15978.018 [file elife-15978-supp1.docx]

| Supplementary file 1. List of primers used in this study | |
| --- | --- |
| **qRT-PCR** |  |
| p21 forward | TGTATATTCAGCATTGTGGGAGGA |
| p21 reverse | CTGGACTGTTTTCTCTCGGCTC |
| miR-34a forward | ACACTCCAGCTGGGTGGCAGTGTCTTAGCT |
| miR-34a reverse | ACTGACTGATGCAATCTCAACTGGTGTCGTGGA |
| miR-139-5p forward | ACACTCCAGCTGGGTCTACAGTGCACGTGTC |
| miR-139-5p reverse | ACTGACTGATGCAATCTCAACTGGTGTCGTGGA |
| GAPDH forward | GATTCCACCCATGGCAAATTC |
| GAPDH reverse | AGCATCGCCCCACTTGATT |
| **ChIP-PCR** |  |
| miR-139 forward | CTCAGGGCTCGGGCTTTAT |
| miR-139 reverse | CCTCCATGCCAGCGTTTCT |
| **Constructs** |  |
| pGL3-miR-139 forward | ccgACGCGTGGCCAGTGGCTGTAACAGAT |
| pGL3-miR-139 reverse | ccgCTCGAGCACCAACTCTTGGACGGTGA |
| pGL3-miR-139-mut forward | GGGAGAGCATGCCAGAAAATGCCTCAGAAAGAAAG |
| pGL3-miR-139-mut reverse | CTTTCTTTCTGAGGCATTTTCTGGCATGCTCTCCC |
| pMIR-PDE4D-WT forward | CTAGTCTGCAGTTTATTTTATTTTATTTTTTGACACAAACTGTAGATTTTAGCAGCCCTGGA |
| pMIR-PDE4D-WT reverse | AGCTTCCAGGGCTGCTAAAATCTACAGTTTGTGTCAAAAAATAAAATAAAATAAACTGCAGA |
| pMIR-PDE4D-mut forward | CTAGTCTGCAGTTTATTTTATTTTATTTTTTctgtgAAtgacatctTTTTAGCAGCCCTGGA |
| pMIR-PDE4D-mut reverse | AGCTTCCAGGGCTGCTAAAAagatgtcaTTcacagAAAAAATAAAATAAAATAAACTGCAGA |
| pSIF-139-Top | GATCCtctacagtgcacgtgtctccagtCTTCCTGTCAGAactggagacacgtgcactgtagaTTTTTG |
| pSIF-139-Bottom | AATTCAAAAAtctacagtgcacgtgtctccagtTCTGACAGGAAGactggagacacgtgcactgtagaG |
